# Sex-Specific Selection Drives the Evolution of Alternative Splicing in Birds

**DOI:** 10.1093/molbev/msaa242

**Published:** 2020-09-25

**Authors:** Thea F Rogers, Daniela H Palmer, Alison E Wright

**Affiliations:** Department of Animal and Plant Sciences, University of Sheffield, Sheffield, United Kingdom

**Keywords:** sex-specific selection, alternative splicing, sexual dimorphism, transcriptome, sexual conflict

## Abstract

Males and females of the same species share the majority of their genomes, yet they are frequently exposed to conflicting selection pressures. Gene regulation is widely assumed to resolve these conflicting sex-specific selection pressures, and although there has been considerable focus on elucidating the role of gene expression level in sex-specific adaptation, other regulatory mechanisms have been overlooked. Alternative splicing enables different transcripts to be generated from the same gene, meaning that exons which have sex-specific beneficial effects can in theory be retained in the gene product, whereas exons with detrimental effects can be skipped. However, at present, little is known about how sex-specific selection acts on broad patterns of alternative splicing. Here, we investigate alternative splicing across males and females of multiple bird species. We identify hundreds of genes that have sex-specific patterns of splicing and establish that sex differences in splicing are correlated with phenotypic sex differences. Additionally, we find that alternatively spliced genes have evolved rapidly as a result of sex-specific selection and suggest that sex differences in splicing offer another route to sex-specific adaptation when gene expression level changes are limited by functional constraints. Overall, our results shed light on how a diverse transcriptional framework can give rise to the evolution of phenotypic sexual dimorphism.

## Introduction

Males and females of many species can have divergent evolutionary optima, and are often subject to conflicting selection pressures (Andersson 1994), yet they share an almost identical set of genes. As a result, when contradictory sex-specific selection pressures act on traits that have a shared genetic basis, significant amounts of sexual conflict can occur (Parker and Partridge 1998; Bonduriansky and Chenoweth 2009). Despite this, sex differences are common across a broad range of phenotypes, including morphology, physiology, behavior, and life history, and it is widely assumed that transcriptional dimorphism encodes these sexually dimorphic traits by breaking down intersexual correlations and facilitating sex-specific adaptation (Connallon and Knowles 2005; Connallon and Clark 2010; Innocenti and Morrow 2010; Mank 2017a). Genes with differences in expression level between males and females are pervasive across many species, and exhibit unique evolutionary properties, including faster rates of sequence and expression evolution (Ranz et al. 2003; Khaitovich et al. 2005; Ellegren and Parsch 2007; Harrison et al. 2015). Indeed, these genes have been the subject of considerable focus in understanding how selection can navigate the constraints imposed by a shared genome, and the consequences for sex-specific adaptation (Mank 2017a, 2017b).

Sex differences in alternative splicing, where different exons are spliced or shuffled in males and females to create distinct sex-specific sequences (Blekhman et al. 2010; Nilsen and Graveley 2010), have the potential to play key roles in sex-specific adaptation, yet they have been largely overlooked with the exception of a few studies (Blekhman et al. 2010; Brown et al. 2014; Gibilisco et al. 2016; Grantham and Brisson 2018). In particular, alternative splicing enables multiple transcripts to be generated from a single gene, increasing sex-specific proteome diversity (Matlin et al. 2005; Nilsen and Graveley 2010). In theory, this could act so that certain exons (e.g., those with sex-specific beneficial functions) are retained in one sex, and certain exons (e.g., those that have sex-specific detrimental effects) are excluded in the other sex, generating distinct sex-specific isoforms. There is mounting evidence that splicing varies substantially across species, sexes, and tissues (Su et al. 2008; Gibilisco et al. 2016), and has important phenotypic consequences for sex determination, disease, physiology, and development (Cline and Meyer 1996; Schütt and Nöthiger 2000; McIntyre et al. 2006; Kalsotra and Cooper 2011; Gerstein et al. 2014). Despite this, although certain isoforms have key cellular roles and mediate important phenotypes, the extent to which global patterns of splicing are functionally relevant is an important point of discussion (Blencowe 2017; Tress et al. 2017a, 2017b; Wan and Larson 2018). Many alternative splicing events are highly tissue-specific and patterns of splicing shift rapidly across species over evolutionary time (Pan et al. 2005; Barbosa-Morais et al. 2012; Merkin et al. 2012; Melé et al. 2015) but whether this reflects stochastic transcriptional noise, relaxed selection, or lineage-specific innovations remains unclear (Blencowe 2017; Tress et al. 2017a, 2017b; Wan and Larson 2018). Importantly, the contribution of sex-specific selection to the rapid turnover of sex differences in splicing across species has yet to be tested, as most studies exploring the link between transcriptional variation and sexual selection have not accounted for sex-specific patterns of alternative splicing.

Furthermore, the factors constraining the evolution of alternative splicing have yet to be investigated. There is growing evidence that pleiotropy, where a gene performs several functions and affects multiple traits, hinders the evolution of gene expression level and limits the response to sex-specific selection (Chen and Dokholyan 2006; Mank et al. 2008; Papakostas et al. 2014). Indeed, genes with broad expression patterns, a proxy for pleiotropy, are less likely to be differentially expressed between males and females (Mank et al. 2008). Alternative splicing could avoid these pleiotropic and functional constraints acting on expression level through the generation of distinct male and female isoforms, thereby acting as an alternate or complementary route to sex-specific adaptation.

Here, we characterize patterns of alternative splicing across males and females of three avian species in order to test the role of sex-specific selection in the evolution of alternative splicing and establish its role in sex-specific adaptation and sexual dimorphism. We identify hundreds of genes that exhibit significant sex-biased alternative splicing and show that sex differences in splicing are correlated with phenotypic sex differences. We find that patterns of sex-specific alternative splicing have evolved rapidly, likely as a product of sex-specific selection, and that genes that are differentially spliced exhibit genomic signatures consistent with sex-specific fitness effects. Broadly, our results provide insight into how, via a diverse transcriptional architecture, the same genome is selected to encode multiple phenotypes, and demonstrates the role of alternative splicing in the evolution of phenotypic complexity.

## Results and Discussion

### Alternative Splicing Is Widespread and Common across Birds

We quantified alternative splicing in males and females across multiple tissues in three avian species that diverged ∼90 Ma (supplementary fig. S1, Supplementary Material online). Splicing was estimated as the relative proportion of two alternative isoforms at each splice site, otherwise referred to as percent spliced-in (PSI). A PSI value of 1 or 0 indicates that only one of the two alternative isoforms is always expressed and a value of 0.5 indicates equal expression of both isoforms. Alternative splicing is common and widespread across all individuals, with an average of 21%, 17%, and 24% of autosomal genes undergoing at least one splice event in the duck, turkey, and guineafowl, respectively (supplementary table S1, Supplementary Material online). We identified five different types of alternative splicing events (supplementary fig. S2, Supplementary Material online); skipped exons (SE), where an exon is either excluded or included from the mRNA, mutually exclusive exons (MXE), where one exon is skipped and the other is retained or vice versa, alternative 5′ and 3′ splice site events (A5′SS and A3′SS), where the exon boundary on either the 5′- or 3′-end of the intron is extended or shortened, and retained intron events, where a whole intron is retained in the final transcript. A gene can exhibit multiple different types of splicing events. SE and MXE splicing events are the most common type of splicing across the three species, with the other types of splicing occurring at very low frequency (supplementary table S1, Supplementary Material online). Additionally, SE and MXE events are also more commonly associated with the generation of functional isoforms than other types of splicing (Weatheritt et al. 2016), and so we focus solely on these in subsequent analyses.

### Tissues Exhibit Distinct Transcriptional Profiles

Next, we examined patterns of sex differences in splicing across tissues. Males and females undergo very similar rates of splicing (supplementary table S1, Supplementary Material online) in both the spleen and the gonad across the autosomes in each of the three species, and this finding is consistent across multiple filtering thresholds (supplementary table S2, Supplementary Material online). However, despite similarities in the total proportion of alternatively spliced genes, patterns of splicing vary substantially between the sexes (table 1 and supplementary table S4, Supplementary Material online).

**Table 1.** Differential Alternative Splicing between Males and Females across Autosomal Splice Sites and Genes.

		Sex-Biased Alternative Splicing Events			Sex-Biased Alternatively Spliced Genes			
Species	Tissue	MXE^a^	SE^b^	Total	MXE^a^	SE^b^	Total	Proportion of Genes
Duck	Gonad	181	677	886	148	551	640	7.6%
Duck	Spleen	7	27	31	6	26	34	0.4%
Turkey	Gonad	91	481	579	78	421	475	5.2%
Turkey	Spleen	2	39	41	2	38	40	0.5%
Guineafowl	Gonad	219	720	977	174	596	701	7.4%
Guineafowl	Spleen	1	13	14	1	13	13	0.1%

a MXE denotes mutually exclusive exon events.

b SE denotes skipped exon events.

**Fig. 1.**
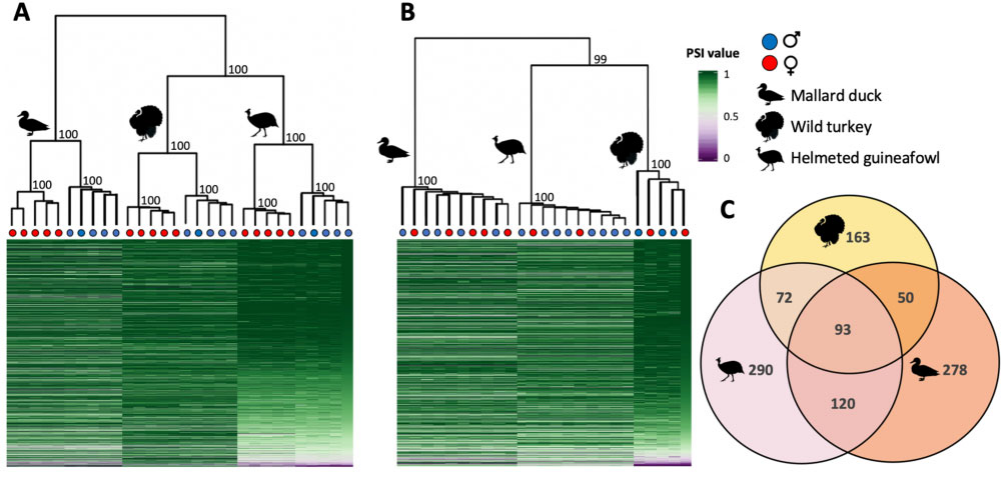
Global patterns of alternative splicing. Panels (*A*) and (*B*) show heatmaps and hierarchical clustering of alternative splicing level in the gonad and spleen, respectively. Percent spliced-in values (PSI) refer to the proportion of alternative isoforms at a splice site, where a PSI value of 1 or 0 indicates that only one of the two alternative isoforms is always expressed and a value of 0.5 indicates equal expression of both isoforms. If a gene undergoes multiple splice events, the average PSI is shown. Numbers on each branch represent the bootstrap probability values. Panel (*C*) shows orthologous genes with significant sex differences in splicing in the gonad that are shared among the duck (pink), turkey (yellow), and guineafowl (orange). We observe significant overlap (*P *<* *0.0001, super exact test) of differentially spliced orthologs across the three species.

Using hierarchical clustering, we found that both gonad and spleen samples cluster first by phylogenetic relatedness, where splicing is more similar between turkey and guineafowl, which diverged ∼30 Ma, than with the duck which diverged ∼90 Ma (supplementary fig. S1, Supplementary Material online). However, in each species, ovary and testis tissue cluster separately whereas the spleen shows no clustering among males and females (fig. 1*A* and *B*). Across all three species, we consistently identified far fewer genes with significant differential alternative splicing in the spleen relative to the gonad (table 1 and supplementary table S4, Supplementary Material online), consistent with results from *Drosophila* (Gibilisco et al. 2016). Our finding that ovaries and testes exhibit distinct transcriptional profiles mirrors patterns of sex differences in expression level (hereafter termed differential expression) across many species (Uebbing et al. 2016), where the gonad often exhibits significant differential expression between males and females for more than half of all expressed genes (Zhang et al. 2007; Mank et al. 2010) but somatic tissues show less differential expression (Yang et al. 2006; Mank et al. 2007; Harrison et al. 2015). This suggests that ovaries and testes are regulated by distinct sex-specific gene regulatory networks, and that sex-specific splice variants play a role in the construction of sex-specific genetic architecture (Mank et al. 2007; Wright et al. 2018). Interestingly, we observe far fewer genes exhibiting differential alternative splicing (3.3%, 1.1%, 2.8% of autosomal genes in the duck, turkey, and guineafowl gonad, respectively; supplementary table S4, Supplementary Material online) relative to differential expression (45.3%, 45.7%, 44.3% in the duck, turkey, and guineafowl gonad, respectively), calling into question the relative effect of splicing versus expression in sex-specific regulatory networks.

### Sex Differences in Alternative Splicing Are Associated with Phenotypic Sexual Dimorphism

We have shown that patterns of splicing vary substantially between the sexes and across tissues (table 1 and supplementary table S4, Supplementary Material online). To test whether this sex-biased transcriptional variation (hereafter termed differential splicing) is associated with phenotypic sex differences, we contrasted patterns of splicing across a gradient of sexual dimorphism. Specifically, we employed contrasts across wild turkey individuals that represent a gradient in male secondary sexual characteristics. The wild turkey exhibits two male phenotypes in the forms of dominant and subordinate males. The species is strongly sexually dimorphic, with dominant males showing greater body size than females, along with a range of sexually selected traits including distinct plumage and mating behaviors (Buchholz 1995, 1997; Hill et al. 2005). Subordinate males develop fewer and less exaggerated sexually selected traits than dominant males, but are clearly male in phenotype, occupying an intermediate position on the continuum of sexual dimorphism.

**Fig. 2.**
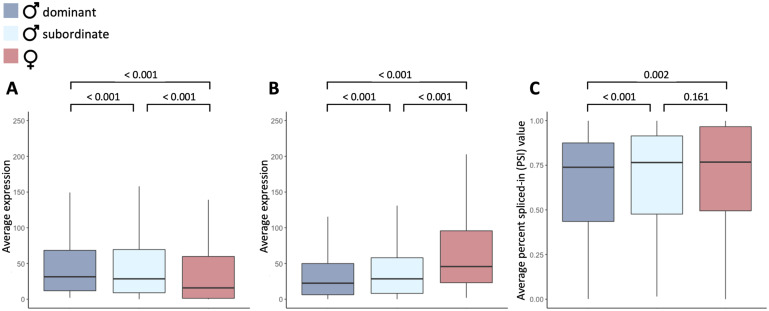
Expression of sex-biased isoforms in dominant male turkeys (dark blue), subordinate male turkeys (light blue), and female turkeys (red). Panel (*A*) and (*B*) show average expression (read counts) of male- and female-biased isoforms, respectively and panel (*C*) is the average percent spliced-in value (PSI) of all sex-biased isoforms. Significance values were calculated using a paired Wilcoxon’s signed-rank test.

Hierarchical clustering of autosomal genes showed that in the gonad, subordinate and dominant males cluster together with high confidence (supplementary fig. S3, Supplementary Material online), and are distinct from females, as opposed to being intersex. However, there were subtle differences in patterns of alternative splicing between dominant and subordinate males (fig. 2). For exons with significant differences in splicing between dominant males and females (table 1), we classified the alternative isoforms as either male- or female-biased depending on whether they were expressed more highly in dominant males or females. We focused our analyses on the gonad as it exhibits the greatest magnitude of differential splicing, making it the tissue most likely to be influenced by sex-specific selection. Subordinate males express male-biased isoforms in the gonad at significantly lower levels than dominant males (paired Wilcoxon signed-rank test, *P* ≤0.001), indicating that patterns of splicing are demasculinized in subordinate males (fig. 2*A*). Subordinate males also express female-biased isoforms at significantly higher levels than dominant males (paired Wilcoxon signed-rank test, *P *<* *0.001) (fig. 2*B*), consistent with feminized splicing. Importantly, subordinate males exhibit intermediate patterns of splicing for all genes that exhibit differential splicing between dominant males and females (fig. 2*C*). These patterns are consistent with the phenotypic sex differences observed across morphs, where subordinate males occupy an intermediate position on the continuum of sexual dimorphism.

We tested whether this pattern was a result of regression toward the mean by randomizing samples 100 times. Each time, we randomly picked three dominant male and three female samples, identified genes with differential splicing, and then assessed the remaining dominant males, females, and subordinate males for the magnitude of splicing (PSI). We found that subordinate males had significantly higher PSI than dominant males for all 100 sample comparisons, and significantly lower PSI than females for the majority of the 100 sample combinations (79 significant comparisons). In contrast, significant differences were observed much less frequently between the randomly chosen dominant male samples (34 significant comparisons) or between female samples (6 significant comparisons), indicating that regression toward the mean is unlikely to explain our results. Gene expression level across turkey morphs has previously been shown to exhibit similar patterns of demasculinization and feminization (Pointer et al. 2013), consistent with a role of transcriptional dimorphism in encoding phenotypic sex differences. Our results suggest a previously overlooked link between genomic and phenotypic dimorphism, where differential alternative splicing works concurrently with differential expression level to produce the diverse transcriptional framework underpinning complex phenotypic sexual dimorphisms.

### Sex-Specific Selection Acts on Isoforms That Are Differentially Expressed between Males and Females

We find that patterns of alternative splicing cluster strongly by species (fig. 1*A* and *B*), consistent with rapid rates of regulatory evolution within lineages. This pattern of clustering is contrary to that observed for gene expression level, including the ones in this study, which clusters first by sex in the gonad, then species (Harrison et al. 2015; Mank 2017a). Our finding that patterns of differential expression are more conserved than patterns of alternative splicing is a broad taxonomic trend (Barbosa-Morais et al. 2012; Merkin et al. 2012; Gibilisco et al. 2016), indicative of rapid turnover of alternative splicing across species. However, we observe significant overlap (*P* < 0.001, super exact test) of differentially alternatively spliced orthologs across the three species (fig. 1*C* and supplementary table S3, Supplementary Material online), indicating that although patterns of splicing evolve quickly, significant sex differences in splicing are limited to a core set of avian genes. To test whether this conserved set of genes is enriched for specific functions, we conducted a Gene Ontology analysis (Mi et al. 2019), but failed to find any significantly enriched terms (*P* < 0.05).

We implemented an evolutionary framework, using regulatory variation as a proxy for selection, to test whether the rapid rate of regulatory evolution we observe is a product of sexual selection. Studies of regulatory variation have recently been implemented as a powerful approach to infer selection (Brawand et al. 2011; Gallego Romero et al. 2012; Moghadam et al. 2012; Dean et al. 2015), where selection on loci increases with expression level (Duret and Mouchiroud 1999; Pál et al. 2001; Drummond et al. 2006; Gout et al. 2010).

Applying this framework to alternative splicing, if purifying selection is the dominant evolutionary force acting on splice variants, we predict highly expressed genes to express fewer isoforms than lowly expressed genes, which might be spuriously transcribed and subject to weaker constraints. Furthermore, when expression level differs between the sexes, purifying selection would be strongest in the sex with the higher expression, resulting in the expression of fewer isoforms in that sex. For example, for male-biased genes, we would predict that males tend to have fewer isoforms than females.

However, if there is sexual selection for sex-specific isoforms, we expect the opposite relationship between isoform diversity and sex. Here, we predict the evolution of novel isoforms to be analogous to gene duplication with neofunctionalization, where the ancestral paralog retains its original function and expression pattern but the newly duplicated paralog evolves sex-specific functions and sex-biased expression (Connallon and Clark 2011). Applying this to splicing, we expect the ancestral splice variant to retain its ancestral expression pattern and function, but the novel sex-specific isoform to evolve sex-specific functions and expression. As a result, we expect a greater diversity of isoforms in the sex with higher expression, where selection for sex-specific isoforms is the greatest. Specifically, males should express more isoforms than females for male-biased genes, where novel male-specific isoforms are free to evolve male-specific functions, whereas isoforms expressed in both sexes are retained to perform their original function. We predict the opposite pattern for female-biased genes, which under sex-specific selection should exhibit a greater diversity of isoforms expressed in females.

These two scenarios generate opposing predictions for the expected patterns of isoform diversity in males and females. To distinguish between these selective regimes, we developed an isoform specificity index ($\tau_{\mathrm{AS}})$ to quantify variation in isoform abundance per gene. This metric is adapted from the tissue specificity index (Yanai et al. 2005), where high values show that a single isoform is always expressed and low values indicate an even representation of multiple isoforms.

**Fig. 3.**
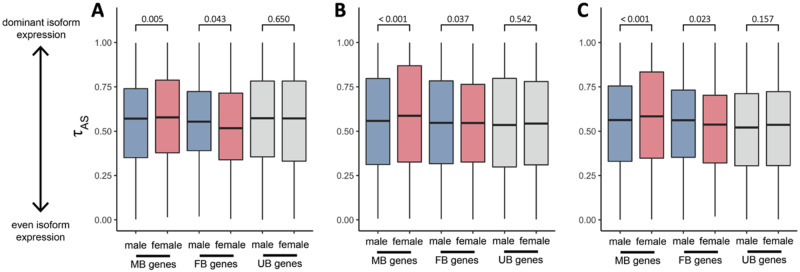
Average male and female isoform specificity ($\tau_{\mathrm{AS}}$) across genes. $\tau_{\mathrm{AS}}$ for genes with male-biased expression level, female-biased expression level, and unbiased expression between the sexes for the (*A*) duck, (*B*) turkey, and (*C*) guineafowl. Significance values were calculated using a paired Wilcoxon’s signed-rank test.

We found a significant relationship between isoform specificity ($\tau_{\mathrm{AS}})$ and expression level across all genes, where highly expressed genes tend to express fewer isoforms than lowly expressed genes (supplementary fig. S5 and table S6, Supplementary Material online). This indicates that purifying selection acts on broad patterns of splicing across the genome, suggesting that global patterns of splicing are functionally relevant (Blencowe 2017; Tress et al. 2017a, 2017b; Wan and Larson 2018). However, we also recovered a significant association with sex, where isoform specificity ($\tau_{\mathrm{AS}})$ differs significantly between males and females for genes that are differentially expressed between the sexes, but not for those with similar expression levels (fig. 3 and supplementary fig. S5, Supplementary Material online). Importantly, this association is reversed between male- and female-biased genes, as we predicted. Specifically, males show significantly greater isoform diversity for male-biased genes, and females show greater isoform diversity for female-biased genes. There are no significant differences in isoform diversity between males and females for unbiased genes. This is consistent with our predictions of selection for sex-specific splice variants, and opposite to what we would expect if purifying selection were the dominant evolutionary force acting on splicing in males and females. These patterns are observed across all three species, which diverged 90 Ma, indicating that the role of sex-specific selection in splicing evolution is a broad taxonomic trend across birds.

If sex-specific isoforms are indeed under selection for sex-specific functions, then we expect these loci to affect fitness differently in males relative to females. To test whether differential splicing has sex-specific effects, we used a population genomic approach across the three avian species, contrasting patterns of intersexual sequence differentiation and balancing selection (Wright et al. 2018). Recent theoretical work has indicated that patterns of elevated intersexual differentiation previously observed in the literature that have been attributed to ongoing sexual conflict would require implausibly large selective pressures and mortality loads (Kasimatis et al. 2017, 2019, 2020; Ruzicka et al. 2020). However, we do not use this approach to infer ongoing conflict, rather, sex-specific genetic architecture which invokes relatively lower genetic loads. Under sex-specific architecture, where loci exhibit sex differences in their phenotypic effects, we predict elevated intersexual differentiation but relaxed balancing selection (Mank 2017b).

Consistent with this prediction, we found that differentially alternatively spliced genes exhibited elevated intersexual *F*_ST_ and low Tajima’s *D* in the duck gonad and guineafowl gonad (χ^2^ test, *P *=* *0.003 and *P *=* *0.059, respectively; supplementary table S5, Supplementary Material online), consistent with differentially spliced genes affecting viability or survival in one sex but having little or no effect in the other. This pattern was not significant in the turkey gonad (χ^2^ test, *P *=* *0.266; supplementary table S5, Supplementary Material online), however, there are many fewer differentially spliced genes in turkey (table 1) which likely limits our power to test for any relationship in this species. Genes that were significantly differentially expressed between males and females were removed from this analysis as they have been shown previously to have sex-specific phenotypic effects (Wright et al. 2018). To confirm that these sex-specific effects are driven by sex-specifically expressed parts of genes, we extracted intersexual *F*_ST_ for sex-biased and unbiased exons. We found that *F*_ST_ was higher across sequences from sex-biased exons relative to unbiased exons in both the turkey and the guineafowl (*P *=* *0.014, *P *=* *0.083, turkey and guineafowl, respectively, paired Wilcoxon signed-rank test) but there was no significant difference in the duck (*P *=* *0.543). This is the first statistical evidence, to our knowledge, that sex-specific selection acts on broad patterns of alternative splicing and that differentially spliced genes across the genome exhibit genomic signatures consistent with sex-specific effects.

### Genes with Sex Differences in Splicing Are Subject to Greater Functional Constraints

**Fig. 4.**
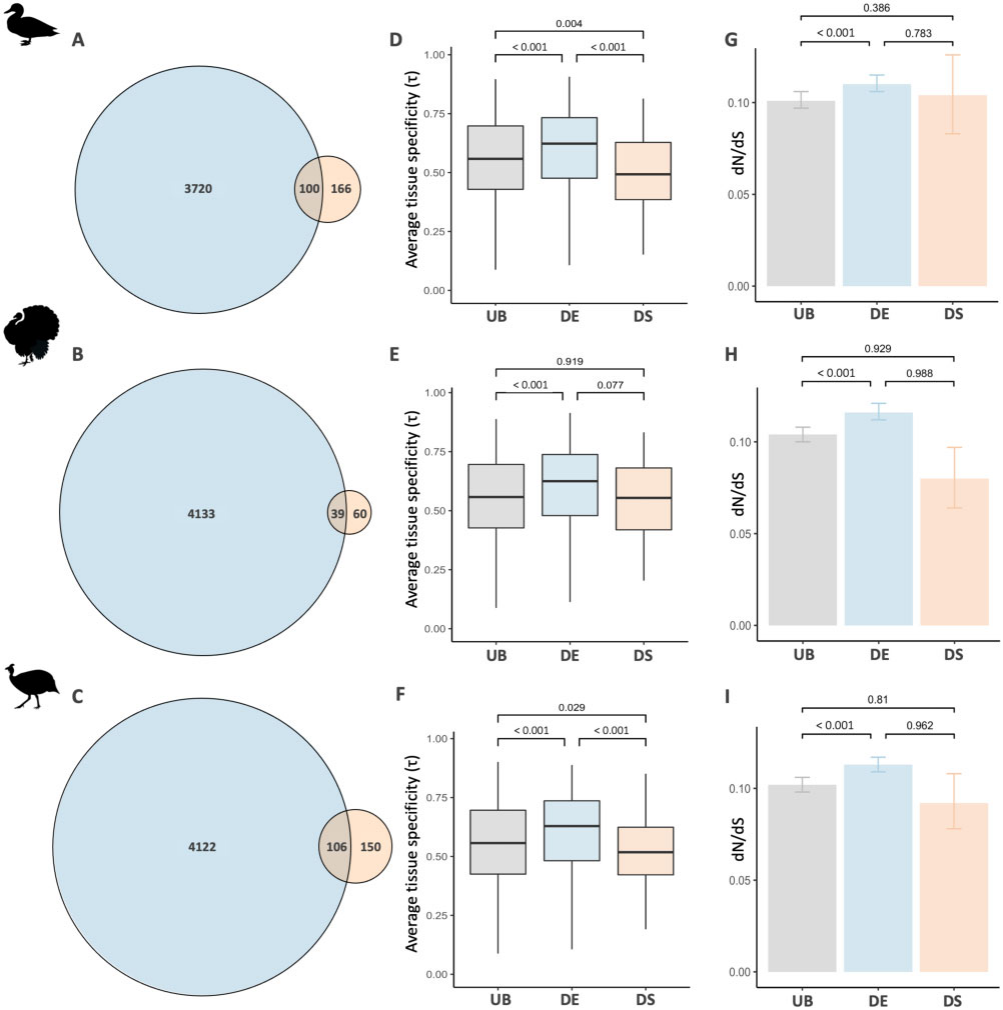
Overlap, tissue specificity, and evolutionary rates of genes with sex differences in splicing and expression level in the duck (*A*, *D*, *G*), turkey (*B*, *E*, *H*), and guineafowl (*C*, *F*, *I*) gonad. Panels (*A*–*C*) show the overlap between differentially spliced (orange) and differentially expressed (blue) genes. Panels (*D*–*F*) show average tissue specificity (*τ*), where 0 denotes genes that are expressed ubiquitously and 1 means genes have tissue-specific expression. Panels (*G*–*I*) show the average ratio of nonsynonymous (d*N*) to synonymous (d*S*) substitutions for genes that are exclusively differentially spliced (orange), exclusively differentially expressed (blue), or unbiased (gray). In (*D*), (*E*), and (*F*), significance values were calculated with Wilcoxon’s rank-sum test. In (*G*), (*H*), and (*I*), 95% confidence intervals and significance values were obtained from 1,000 bootstrap replicates.

Pleiotropy is thought to hinder the evolution of differential gene expression level and limit the response to sex-specific selection (Mank et al. 2008; Meisel 2011). Indeed, genes with broad expression patterns, a proxy for pleiotropy, are less likely to be differentially expressed (Mank et al. 2008). Alternative splicing might avoid pleiotropy and other constraints acting on expression level through the generation of distinct male and female isoforms. If so, we expect differential alternative splicing to be more common in genes with similar expression patterns between males and females. In line with our prediction, we found that while nonsignificant (duck *P *=* *0.06, turkey *P *=* *0.55, guineafowl *P *=* *0.49, hypergeometric tests with Benjamini–Hochberg correction), there is less overlap than expected between differentially expressed and differentially spliced genes in the gonad (RF<1; duck RF=0.83, turkey RF=0.86, guineafowl RF=0.94, fig. 4*A*–*C*, and supplementary tables S7 and S8, Supplementary Material online). These results are consistent across multiple filtering thresholds and types of splicing events (supplementary table S8, Supplementary Material online).

Next, we explicitly tested whether genes under functional constraints are more predisposed to evolve differential splicing. First, we calculated a measure of tissue specificity (*τ*), a proxy for pleiotropy, where lower values indicate even expression distribution across tissues and larger values equate to greater levels of tissue specificity (Yanai et al. 2005). Measurements of *τ* were derived from the chicken UniGene database (Mank et al. 2008) and encompass expression patterns from nine tissues. Across all three species, we found that differentially spliced genes have significantly broader expression patterns relative to genes that are unbiased in expression, consistent with greater functional constraint (fig. 4*D*–*F*). This is in stark contrast to genes with differential expression level which, as previously observed (Mank et al. 2008; Meisel 2011), have greater tissue specificity than unbiased genes. Second, we employed contrasts of coding sequence evolution between genes that are unbiased, are exclusively differentially spliced or exclusively differentially expressed. Previously, differentially expressed genes have been shown to exhibit elevated rates of coding sequence evolution in a wide range of species as a consequence of relaxed evolutionary constraint and genetic drift (Gershoni and Pietrokovski 2014; Harrison et al. 2015). In contrast, we find that genes with differential splicing do not exhibit significantly elevated rates of sequence evolution in comparison to unbiased genes or genes that are differentially expressed between the sexes (fig. 4*G*–*I*), consistent with stronger purifying selection acting on coding sequences. This pattern is conserved when accounting for gene length and expression level, although the pattern then becomes nonsignificant in the duck (supplementary table S9, Supplementary Material online). Taken together, these results suggest that when genes are subject to functional constraints, the evolution of sex-specific isoforms may offer a more viable mechanism than changes in expression level to achieve sex-specific functions.

## Concluding Remarks

Our results indicate that sex-specific selection acts on broad patterns of alternative splicing across the genome, which in turn may facilitate the evolution of sexually dimorphic phenotypes. Sex differences in alternative splicing and gene expression level are restricted to distinct sets of genes, where differential alternative splicing is limited to genes subject to strong purifying selection and functional constraint, indicating that splicing may function as an alternate route to sex-specific adaptation. However, it remains unclear whether dimorphism is a consequence of aggregate patterns of sex-biased splicing or large-effect loci, or how the magnitude of splicing scales with phenotypic differences. Taken as a whole, our findings demonstrate how diverse patterns of transcriptional regulation can play an important role in phenotypic complexity.

## Materials and Methods

### Quality Filtering and Mapping

Previously, we obtained tissue samples, extracted, and sequenced RNA from semicaptive populations of the mallard duck (*Anas platyrhynchos*), wild turkey (*Meleagris gallopavo*), and helmeted guineafowl (*Numida meleagris*) (BioProject ID PRJNA271731, Harrison et al. 2015). The duck diverged from the guineafowl and turkey ∼90 Ma, and the turkey and guineafowl diverged 30 Ma, providing medium- and long-term evolutionary comparison points for assessing divergence in splicing (supplementary fig. S1, Supplementary Material online). This includes RNA-seq data from five male and five female individuals of each species except for the turkey, where five dominant male, two subordinate male, and five female gonad samples were taken along with three dominant male and two female spleen samples. RNA data were quality filtered using Trimmomatic v0.36 (Bolger et al. 2014). We filtered reads containing adapter sequences and trimmed reads if the sliding window average had a Phred score over four bases that was <15 or if the leading/trailing bases had a Phred score <3. The program used to quantify alternative splice events, rMATS (Shen et al. 2014), requires all reads to be equal length so reads were removed postfiltering if either read pair was <95 bp in length and all remaining reads were trimmed to 95 bp.

RNA-seq reads were mapped to respective reference genomes obtained from Ensembl (mallard duck; CAU_duck1.0; GCA_002743455.1, wild turkey; Turkey_2.01; GCA_000146605.1, helmeted guineafowl; NumMel1.0; GCA_002078875.2), using HISAT2 v.2.10 (Kim et al. 2015). We suppressed discordant and unpaired alignments for paired reads and excluded reads from the SAM output that failed to align. Reported alignments were tailored for transcript assemblers including StringTie. These alignments were used in downstream analyses to quantify both alternative splicing and gene expression levels to ensure accurate comparisons between patterns of splicing and gene expression levels.

### Quantifying Alternative Splicing

We quantified alternative splicing in males and females in each species using rMATS v.4.0.3. Specifically, rMATS assesses annotated splice junctions in the reference genome for alternative splicing and detects differential splicing between two groups of samples. Splicing at each splice site is measured as the PSI, which indicates the proportion of two alternative isoforms at each splice site. A PSI value of 1 or 0 indicates that only one of the two alternative isoforms is always expressed and a value of 0.5 indicates equal expression of both isoforms. We detected alternative splicing events using 0 < PSI<1 in more than half of the individuals in each sample group. To compare splicing between groups of samples, rMATS calculates an inclusion difference (ΔPSI) (average PSI of male samples−average PSI of female samples), which ranges from 1 (the one isoform is only expressed in males) to −1 (the alternative isoform is only expressed in females). Therefore, ΔPSI of 0 means that patterns of splicing do not differ between males and females (i.e., the proportion of alternative isoforms for that splice site is the same between the sexes). rMATS uses a likelihood-ratio test to identify significant differences in ΔPSI between males and females. We identified differential splicing events using an FDR *P* value <0.05 and ΔPSI threshold of 0.1 following Grantham and Brisson (2018). The only exception was for analyses comparing patterns of differential splicing to differential expression where we used an FDR *P* value <0.05 and male: female log_2_-fold change PSI value of 1 to ensure equivalent thresholds were implemented. We calculated the significance of the overlap between differentially spliced orthologs using the SuperExactTest package (Wang et al. 2015) in R. Patterns of splicing were only quantified for autosomal genes as the Z chromosome is subject to unusual patterns of sex-specific selection due to its unequal inheritance pattern between males and females (Rice 1984). This workflow is summarized in supplementary figure S4, Supplementary Material online.

It has been suggested that many of the splicing events detected through next-generation sequencing approaches reflect stochastic transcriptional noise, however, this has been the subject of considerable recent debate (Melamud and Moult 2009; Tress et al. 2017a, 2017b; Wan and Larson 2018). We implemented a number of stringent filters to remove alternative splicing events that are likely nonfunctional noise. First, we evaluated splicing using only reads mapping to exon–exon boundaries that span splicing junctions to quantify splicing. Second, following Grantham and Brisson (2018), splicing sites were excluded if the number of reads supporting the inclusion and spliced exon junction was <20 in at least half the samples of both sexes in each tissue separately. Finally, although rMATS analyses different types of alternative splicing events, SE and MXE events are more commonly known to translate into functional isoforms (Weatheritt et al. 2016). These types of splicing comprise the majority of splice events we identified (supplementary table S1, Supplementary Material online) and so subsequent analyses were only performed on SE and MXE splicing events.

### Cluster Analysis of Alternative Splicing Data

We assessed transcriptional similarity of splicing across samples, as measured by PSI, using the R package Pvclust (Suzuki and Shimodaira 2006). Hierarchical clustering with Euclidean distance was performed and the reliability of each of the trees produced was tested by bootstrap resampling (1,000 replicates).

### Quantifying Gene Expression Level

SAM files generated from HISAT2 were coordinate sorted using SAMtools v1.9 (Li et al. 2009) and converted to BAM format. For each species, StringTie v1.3.5 (Pertea et al. 2015) was used to estimate gene expression level only for transcripts in the reference genome, ignoring novel transcripts, to ensure that expression was quantified for the same set of loci across all samples. We then extracted read count information directly from the StringTie output to generate count matrices for genes and transcripts as recommended by the StringTie pipeline. To ensure that our estimates of expression level were not biased by differences in alternative splicing across samples, we calculated gene expression level using only constitutively expressed exons (i.e., removing exons that are alternatively spliced or differentially alternatively spliced between males and females, FDR<0.05).

In each species, a minimum expression level threshold of 1 log CPM in at least half of the individuals of both sexes was imposed to remove lowly expressed genes in the gonad and spleen separately. Expression level was normalized using TMM (trimmed mean of *m* values) in EdgeR (Robinson et al. 2010). Genes were excluded from the analysis if they were single-exon or not located on annotated autosomal chromosomes. Sex-biased genes were identified using a standard log_2_-fold change value of 1 and FDR *P* value <0.05 (Assis et al. 2012; Harrison et al. 2015). This workflow is summarized in supplementary figure S4, Supplementary Material online.

### Estimating Isoform Specificity ($\tau_{\mathrm{AS}}$)

We developed an isoform specificity index to quantify variation in isoform abundance per gene. This is adapted from the tissue specificity index (*τ*) (Yanai et al. 2005), a commonly used metric that calculates whether expression is broadly expressed or localized in one tissue. Here, we instead use expression of each isoform to calculate isoform specificity, where a value of 0 indicates an even representation of isoform abundance and a value of 1 shows that a single isoform is always expressed. We call this measure $\tau_{\mathrm{AS}}$. For a given gene, $\tau_{\mathrm{AS}}$ is defined as:

$$\tau_{\mathrm{AS}}=\;\frac{\sum_{i=1}^{n}\left(1-{\hat{x}}_{i}\right)+(1-{\hat{y}}_{i})}{n-1};\;{\hat{x}}_{i}=\;\frac{x_{i}}{\underset{1\leq i\leq n}{\max}\left(x_{i},y_{i}\right)},\;{\hat{y}}_{i}=\;\frac{y_{i}}{\underset{1\leq i\leq n}{\max}\left(x_{i},y_{i}\right)},\;$$

where n is the total number of isoforms (assuming each splice site produces two isoforms), $x_{i}$ is the read count supporting the inclusion of the exon in the gene product, and $y_{i}$ is the read count supporting the exclusion of the exon from the gene product. We excluded splice sites that did not pass the coverage thresholds described above, and we excluded any exon that did not have a minimum read count of 20 in at least half of the individuals (within or between sexes) supporting both inclusion and exclusion of the exon. We then calculated male and female $\tau_{\mathrm{AS}}$ for each gene. Importantly, power to detect isoform variation is limited by expression level so we reduced read counts in the sex with higher expression before calculating $\tau_{\mathrm{AS}}$. Specifically, read counts in the more highly expressed sex were scaled to the sex with the lower expression for each gene. This accounts for reduced power to detect splice events in samples with lower expression. In addition to this, to check that our results were not biased by variation in sequencing depth across samples, we normalized $\tau_{\mathrm{AS}}$, where read counts were divided by total library size in each sample. We tested for statistical differences between male and female $\tau_{\mathrm{AS}}$ using a paired Wilcoxon’s signed-rank test.

### Estimating Population Genomic Statistics

For each individual, we merged spleen and gonad BAM files using SAMtools v1.9 (Li et al. 2009) with the exception of the turkey, where both tissues were not sequenced for all individuals so we used only gonad data for subsequent analyses. We used ANGSD (Korneliussen et al. 2014) to estimate population genetic summary statistics, following our previous approach (Wright et al. 2018, 2019) as ANGSD implements methods to account for uneven sequencing depth and is therefore appropriate for transcriptome data. We filtered BAM files to discard reads if they did not uniquely map, had a flag ≥256, had a mate that was not mapped, or had a mapping quality <20. Bases were filtered if base quality was <13 or if there were data in fewer than half the individuals. Mapping quality scores were adjusted for excessive mismatches and quality scores were adjusted around indels to rule out false single-nucleotide polymorphisms. We identified and removed related individuals (two wild turkey samples) from our analyses using NGSRELATE (Korneliussen and Moltke 2015) to avoid violating Hardy–Weinberg assumptions.

We calculated sample allele frequency likelihoods at each site from genotype likelihoods with the SAMtools model in ANGSD. Next, we estimated the overall unfolded site frequency spectrum for each species (Nielsen et al. 2012). Specifically, at each site we randomly sampled an allele frequency according to its likelihood, as calculated by ANGSD. Finally, we computed genetic diversity indices, including allele frequency posterior probability and Tajima’s *D* using the site frequency spectrum as prior information with ANGSD thetaStat (Korneliussen et al. 2014).

Intersexual *F*_ST_ was calculated using the same procedure and filtering criteria as above except that we filtered out bases where we had data in fewer than half the individuals in males and females separately. We quantified Hudson’s *F*_ST_, which is less sensitive to small sample sizes (Bhatia et al. 2013; Gammerdinger et al. 2020). Estimates across coding regions of autosomal loci were obtained using weighted averages, where per‐gene *F*_ST_ is the ratio between the sum of the between‐populations variance across loci and the sum of the total variance across loci.

Immunity genes can generate patterns of balancing selection via mechanisms such as heterozygote advantage (Stahl et al. 1999; Rockman et al. 2010; Hedrick 2011) and negative-frequency dependent selection (Croze et al. 2016). Therefore, genes with potential immune function were excluded from the population genomic analyses. Specifically, we removed all loci with the terms “immune” or “MHC” in their Gene Ontology annotations from population genomic analyses. Furthermore, we applied a strict minimum expression level threshold of 2 log CPM in at least half of the individuals of both sexes to remove lowly expressed genes that may bias population genomic analyses.

### Testing the Overlap between Differentially Spliced and Expressed Genes

We tested whether differentially spliced genes are also differentially expressed. First, we estimated the expected number of genes that are both differentially spliced (DSG) and differentially expressed (DEG) as (total no. DSG × total no. DEG)/total no. expressed genes. Next, we calculated the representation factor (RF), which is the observed number of overlapping genes divided by the expected number. If RF < 1, there is less overlap between differentially spliced and expressed genes than expected and RF > 1, there is more overlap than expected. We tested whether the overlap was significantly less than expected using the hypergeometric test with the phyper function in R. We calculated adjusted *P* values using the Benjamini–Hochberg (FDR) correction.

### Identifying Orthologous Genes across Species

Coding sequences were downloaded from Ensembl v98 (Zerbino et al. 2018) for the mallard duck (*A. platyrhynchos*; CAU_duck1.0; GCA_002743455.1), wild turkey (*M. gallopavo*; Turkey_2.01; GCA_000146605.1), helmeted guineafowl (*N. meleagris*; NumMel1.0; GCA_002078875.2), and zebra finch (*Taeniopygia guttata*; taeGut3.2.4). The longest isoform was retained for each species, and reciprocal orthologs across the four taxa were identified using BlastN v2.9.0+ (Altschul et al. 1990) with an e-value cutoff of 1 × 10^−10^ and minimum percentage identity of 30%. Across the duck, turkey, guineafowl, and zebra finch, 10,622 reciprocal orthologs were identified. We also identified pairwise reciprocal orthologs with the chicken (*Gallus gallus*) for the duck, turkey, and guineafowl using the same approach. This resulted in 13,425, 12,764, and 13,942 orthologs in the duck, turkey, and guineafowl, respectively.

### Estimating Isoform Specificity (*τ*)

Tissue specificity (Yanai et al. 2005) was calculated from the chicken UniGene database, as previously described (Mank et al. 2008), and encompasses expression level patterns from nine tissues. Lower values indicate even expression level distribution across tissues and larger values equate to greater levels of tissue specificity. For each species, we extracted tissue specificity for genes with pairwise reciprocal orthologs in the chicken, resulting in *τ* values for 4,747, 5,131, and 5,200 genes in the duck, turkey, and guineafowl, respectively.

### Estimating Rates of Coding Sequence Evolution

Orthologous sequences were aligned with PRANK v.140603 (Löytynoja and Goldman 2008), using a previously published phylogeny (Harrison et al. 2015). The sequence alignments were then checked for gaps, and for poorly aligned regions using SWAMP v.31-03-14 (Harrison et al. 2014) with a threshold of 4 in a window size of 5 bases and a minimum sequence length of 75 bp. Evolutionary parameters were estimated using the branch model in PAML v.4.8a (Yang 2007). Orthologous genes with d*S*>2 were filtered from subsequent analyses as this represents the point of mutational saturation in avian sequence data (Axelsson et al. 2008; Harrison et al. 2015). We extracted the number of nonsynonymous sites (*N*), the number of nonsynonymous substitutions (*N*d*N*), the number of synonymous sites (*S*), and the number of synonymous substitutions (*S*d*S*) for each taxon in order to calculate d*N*/d*S* weighted by alignment length (Mank et al. 2010; Harrison et al. 2015). We then generated 1,000 bootstrap replicates to obtain 95% confidence intervals and tested for significant differences between gene categories using 1,000 permutations. We tested if the pattern of d*N*/d*S* was conserved after controlling for gene length and gene expression level using multiple regression and an ANOVA test implemented in R. 

## Supplementary Material

Supplementary data are available at *Molecular Biology and Evolution* online.

## Supplementary Material

msaa242_Supplementary_Data
