# Supplementary material for: Sex-Specific Selection Drives the Evolution of Alternative Splicing in Birds
Source: Mol Biol Evol. 2020 Sep 25;38(2):519–30. doi: 10.1093/molbev/msaa242 (PMC7826194; doi:10.1093/molbev/msaa242)
Supplement: msaa242_Supplementary_Data [file msaa242_supplementary_data.zip › msaa242-suppl_data/Supplementary Info.pdf]

SUPPLEMENTARY INFORMATION

TABLES

Table S1. Number of autosomal alternative splicing events and alternatively spliced genes in each species and tissue. Alternative splicing events are defined as those with 0 < PSI < 1 and a minimum of 20 reads spanning the splice junction in more than half the individuals in each sample. Genes were classified as alternatively spliced if they had at least one alternative splicing event. M and F denote males and females respectively. We categorized alternative splicing events as one of five splice types; skipped exons (SE), mutually exclusive exons (MXE), alternative 5′ and 3′ splice sites (A5′SS and A3′SS), and retained intron (RI) events.

Table S2. The total proportion of alternatively spliced autosomal genes in each species and tissue under different filtering parameters. The read count filter column refers to the total reads required to support the splice junction in more than half the individuals in each sample.

Table S3. List of zebra finch, chicken, mallard duck, wild turkey and helmeted guineafowl orthologs expressed in the gonad of the three species studied, and whether they are sex-biased (SB) or unbiased (UB) in splicing.

Table S4. Number of autosomal alternative splicing events and alternatively spliced genes in each species and tissue under different filtering parameters. The read count filter column refers to the total reads required to support the splice junction in more than half the individuals in both males and females. We defined sex-biased splicing from significant differences in percent spliced-in (PSI) between males and females (FDR p-value <0.05). We then imposed two different filtering thresholds based on the magnitude of sex differences in PSI. We used a Δ PSI (average PSI of male samples – average PSI of female samples) threshold of 0.1 following previous approaches (Grantham & Brisson MBE 2018). We also used a male: female $\log_2$ fold change PSI value of 1 for analyses comparing splicing and expression to ensure equivalent thresholds were implemented. We categorized alternative splicing events as one of five splice types; skipped exons (SE), mutually exclusive exons (MXE), alternative 5′ and 3′ splice sites (A5′SS and A3′SS), and retained intron (RI) events.

Table S5. Distribution of F_{ST} and Tajima's D (TD) for each bird species. Only autosomal genes expressed in the gonad were included in the analyses. We split the data into three quantiles to define high F_{ST} and low Tajima's D.

Table S6. Results of multi-predictor model to test for the relationship between isoform diversity, expression level and sex (isoform diversity ~ expression level + sex).

Table S7. List of differentially expressed (DE) genes and differentially alternatively spliced (DS) genes in each species.

Table S8. Hypergeometric tests for whether the overlap between differentially spliced and expressed genes was significantly less than expected across different splicing events and filtering thresholds. Sex-biased splicing is defined using significant differences in percent spliced-in (PSI) between males and females (FDR p-value <0.05). We also used a male: female

log_2 fold change PSI value of 1 or 0 for analyses comparing splicing and expression to ensure equivalent thresholds were implemented.

Table S9. The effect of sex-bias on the ratio of nonsynonymous (dN) to synonymous (dS) substitutions (dN/dS) for unbiased (UB), differentially expressed (DE) and differentially spliced (DS) genes after controlling for gene length (bp) and gene expression level (logCPM) using multiple regression. Estimates and p-values for DE and DS genes relative to UB genes are from a multiple linear regression (dN/dS ~ sex bias + gene length + gene expression level). The F values are calculated using analysis of variance (ANOVA) along with p-values for the categorical variable.

FIGURES

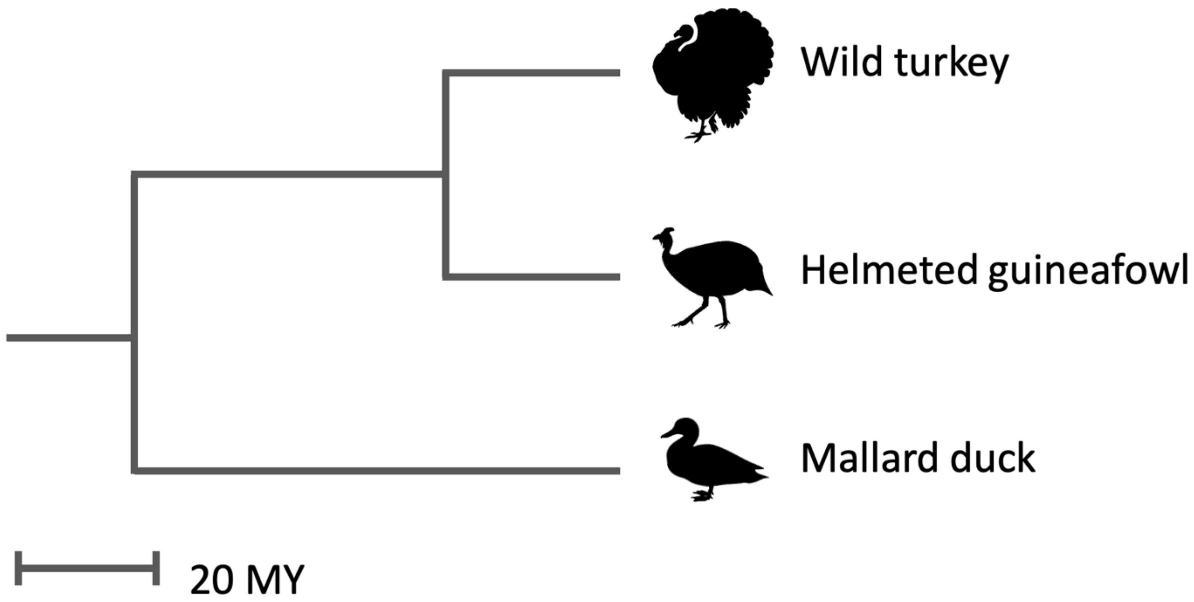

Fig. S1. Phylogenetic relationships across the three avian species used in this study. The mallard duck, wild turkey and helmeted guineafowl shared a common ancestor approximately 90 million years ago.

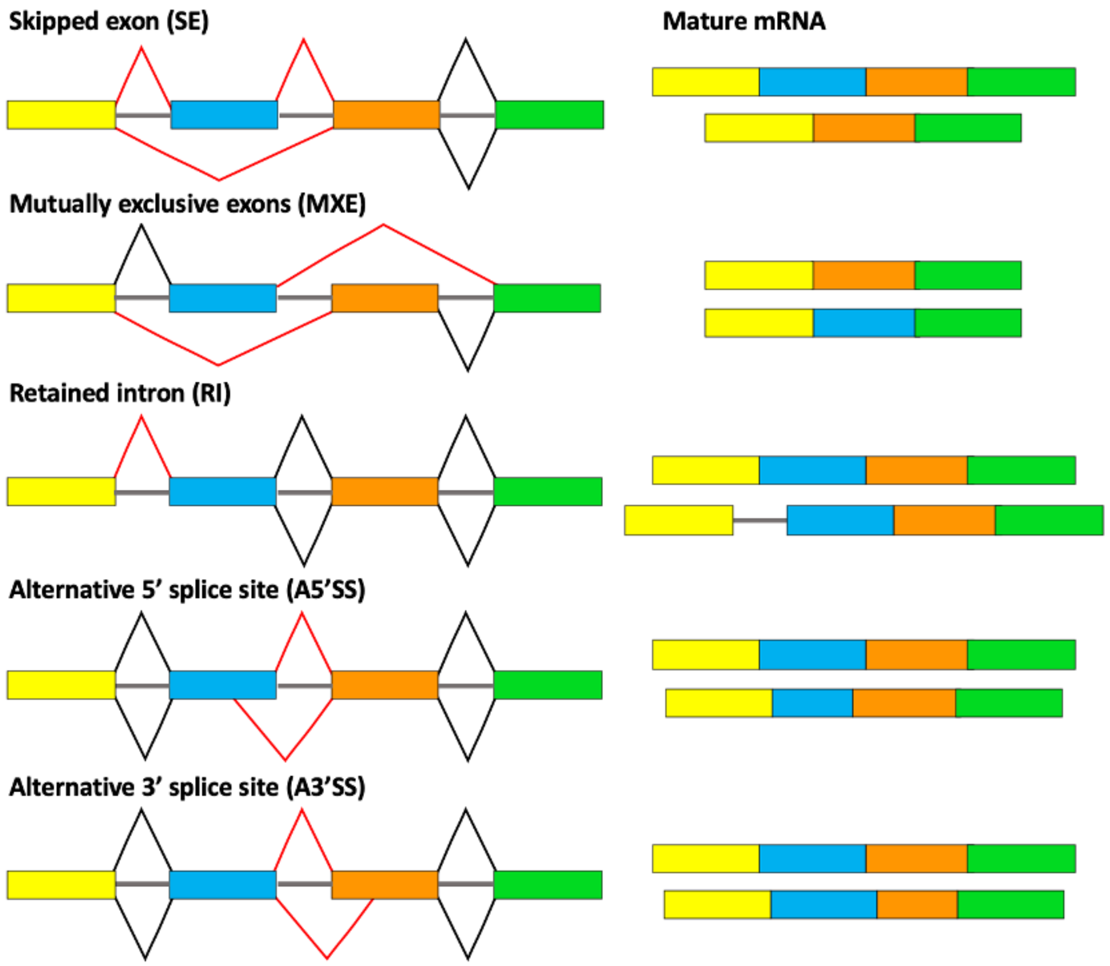

Fig. S2. Schematic diagram of the different types of alternative splicing events. Exons are represented by coloured boxes and introns by grey lines. Lines connecting exons represent regions that are removed from the final transcript. Red lines indicate the two alternative splice events.

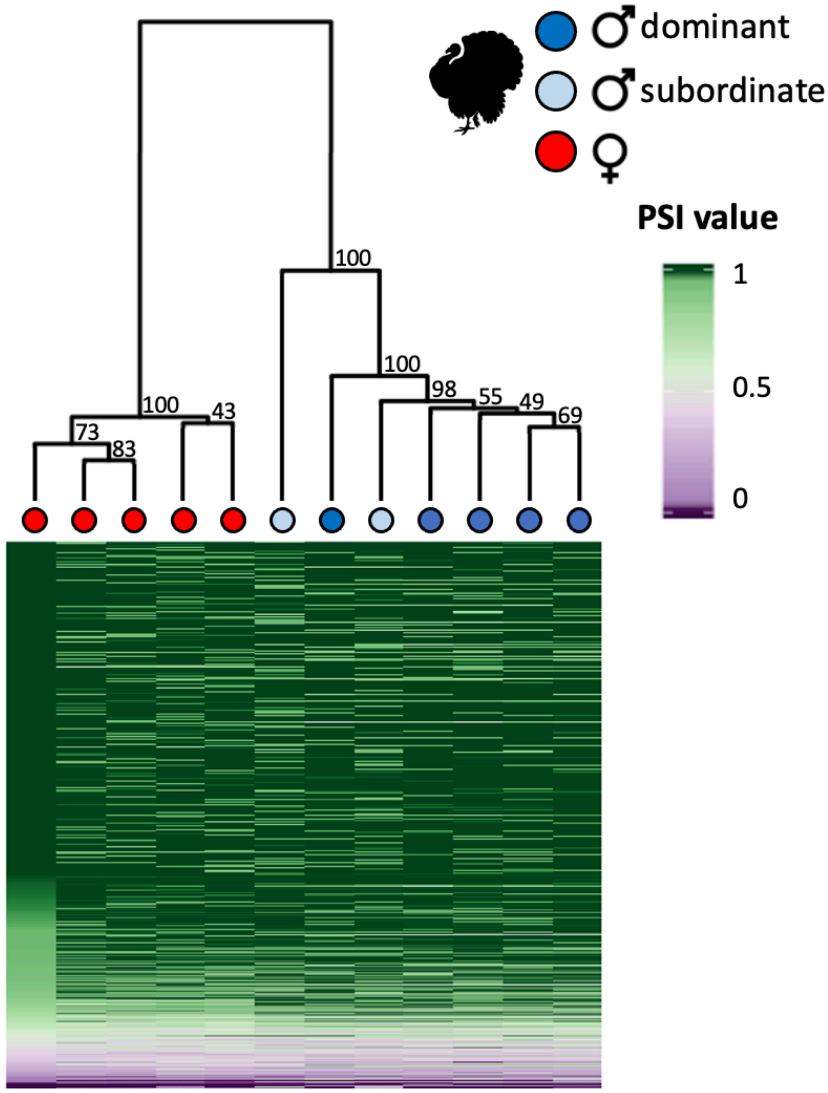

Fig. S3. Heatmap and hierarchical clustering of alternative splicing in the gonad across dominant male, subordinate male and female turkey individuals. Percent spliced-in values (PSI) refer to the proportion of long to short isoforms of each splice site expressed per sample. Green depicts greater inclusion of the long isoform. If a gene undergoes multiple splice events, the average PSI is shown. Numbers on each branch represent the bootstrap probability values. Only autosomal genes are included in the analysis.

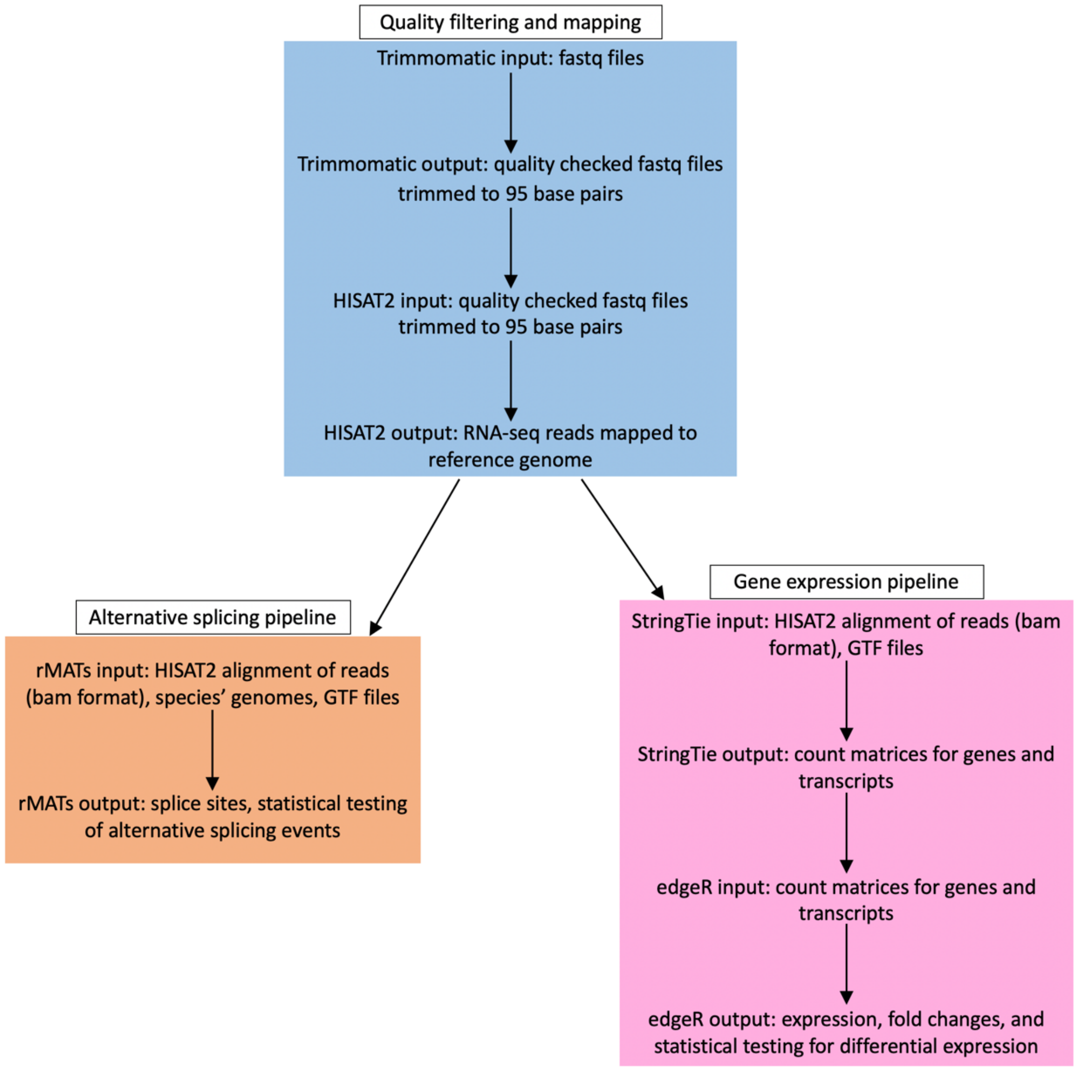

Fig. S4. Workflow to identify alternative splicing events and differences in gene expression level in this study.

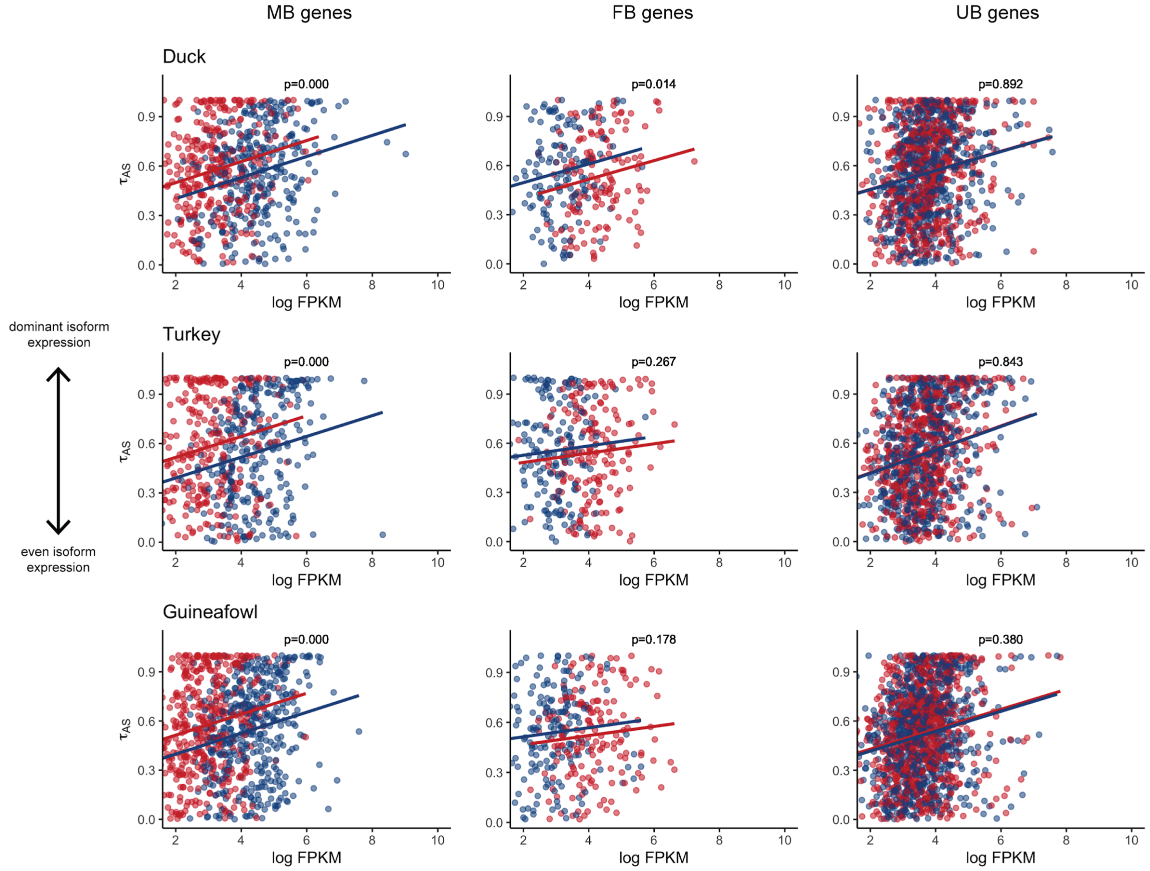

Figure S5: Linear regressions of τ_{AS} over expression level and sex for male-biased (MB), female-biased (FB), and unbiased (UB) genes for duck, turkey, and guineafowl. Blue lines represent male values and red lines represent female values. Each p value denotes the statistical significance of male versus female intercepts.
